# High-Frequency Optical Coherence Elastography for Gingival Tissue Characterization: Variability in Stiffness and Response to Physiological Conditions

**DOI:** 10.34133/bmr.0044

**Published:** 2024-07-01

**Authors:** Wonjoon Moon, Xu Feng, Guo-Yang Li, Seok-Hyun Yun

**Affiliations:** ^1^ Harvard Medical School and Wellman Center for Photomedicine, Massachusetts General Hospital, Boston, MA 02114, USA.; ^2^ Harvard-MIT Division of Health Sciences and Technology, Cambridge, MA 02139, USA.

## Abstract

Accurate measurement of gingiva’s biomechanical properties in vivo has been an active field of research but remained an unmet challenge. Currently, there are no noninvasive tools that can accurately quantify tensile and shear moduli, which govern gingival health, with sufficiently high accuracy. This study presents the application of high-frequency optical coherence elastography (OCE) for characterizing gingival tissue in both porcine models and human subjects. Dynamic mechanical analysis, histology studies, and strain analysis are performed to support the OCE result. Our findings demonstrate substantial differences in tissue stiffness between supra-dental and inter-dental gingiva, validated by dynamic mechanical analysis and OCE. We confirmed the viscoelastic, nearly linear, and transverse-isotropic properties of gingiva in situ, establishing the reliability of OCE measurements. Further, we investigated the effects of tissue hydration, collagen degradation, and dehydration on gingival stiffness. These conditions showed a decrease and increase in stiffness, respectively. While preliminary, our study suggests OCE’s potential in periodontal diagnosis and oral tissue engineering, offering real-time, millimeter-scale resolution assessments of tissue stiffness, crucial for clinical applications and biomaterial optimization in reconstructive surgeries.

## Introduction

Periodontal diseases, impacting the gingiva, are prevalent inflammatory conditions affecting 20% to 50% of the global population [1]. While accurate diagnosis is important for early treatment and preventing irreversible tissue loss [2], periodontal diseases present diagnostic challenges. Periodontitis, the advanced stage, is marked by noticeable symptoms including redness, texture changes, swelling, and bleeding [3]. In contrast, early-stage gingivitis often exhibits subtle, painless symptoms, making detection difficult. Current diagnostic techniques depend heavily on clinical expertise, which can lead to errors.

The biomechanical properties of gingival tissues, heavily influenced by collagen fibers and water content, are essential for understanding periodontal health [4–7]. Inflammation-induced changes in the gingiva, such as collagen degradation and swelling, significantly alter these properties [4,8,9]. These observations underscore the potential to tissue elasticity as an indicator of inflammation. However, measuring gingival tissue elasticity in vivo poses significant challenges. Traditional methods like tensile testing, dynamic mechanical analysis (DMA), and atomic force microscopy have limitations, especially in natural physiological conditions [10–15].

This study introduces the use of optical coherence elastography (OCE) for characterizing gingival tissue. OCE, a noninvasive technique that measures tissue stiffness through imaging of mechanical waves, has shown promise in biomechanical characterizations, particularly in ophthalmology [16,17]. We adapted advanced, wave-based OCE [18] for both ex vivo porcine and in vivo human gingival tissues, achieving high accuracy and spatial resolution. Our findings indicate marked differences in stiffness across various gingival regions and under physiological states. This study not only confirms OCE’s efficacy in assessing biomechanical properties of gingival tissues but also highlights its potential utility in broader periodontal research and clinical applications.

## Materials and Methods

### Porcine gingiva samples

We utilized fresh porcine mandibles obtained 1 h post-mortem, as well as frozen porcine mandibles that were frosted 1 h post-mortem (provided by Research 87 Inc., Boylston, MA, USA). Each mandible, containing 4 incisors, was sectioned using a saw. During experiments, the sample was maintained atop a gauze pad moistened with phosphate-buffered saline (PBS), which was situated within a petri dish. The tissue surface being measured was always oriented upwards, avoiding direct contact with the PBS-soaked gauze. During the measurements, the samples were regularly rehydrated with PBS to maintain tissue integrity. For the extraction of full-thickness gingiva tissue from the bone, a combination of a scalpel and dental elevator was employed. In the hydration model, the target gingiva was covered with gauze soaked in PBS. The samples were then placed in a petri dish at room temperature. To preserve humidity, the bottom of the petri dish was also lined with PBS-soaked gauze. This procedure was replicated for the collagen degradation model, substituting the PBS with a trypsin solution (TrypLE 1X, Gibco, USA). For the dehydration model, gingival tissues were exposed to air at room temperature, allowing them to dry naturally. All measurements for each sample were conducted within a single day, ensuring that no samples were reused on subsequent day.

### Histology

The gingival tissues were first fixed in 10% formalin for 48 h, then stained with hematoxylin and eosin (H&E) or Masson trichrome to facilitate detailed observation. We prepared cross-sections of the tissues with a thickness of 5 μm for microscopic examination. These prepared slides were examined under a microscope at 4× magnification. The measurement of tissue thickness was performed using ImageJ software. To obtain a panoramic view of the overall anatomy, teeth were carefully extracted from the mandible using dental forceps. Subsequently, the complete gingiva surrounding the 4 incisors was used in its intact form for analysis. For specific comparative studies between different gingival positions, the full-thickness labial gingiva from each designated position was meticulously excised from the bone.

### Dynamic mechanical analysis

We obtained porcine gingiva samples from various locations, each carefully excised in full thickness from the bone. These samples were then trimmed to rectangular shapes, approximately 3 mm in width and 4 mm in length. The dynamic mechanical properties of the samples were analyzed using a dynamic mechanical analyzer (DMA1, Mettler Toledo, Switzerland) in compression mode. Initially, the device automatically determined the appropriate preload for each sample. A displacement scan was then conducted to identify the displacement amplitude that resulted in a stable complex modulus. Following this, we performed a frequency scan using a fixed displacement amplitude of 30 μm. The frequency range for these scans varied between 1.7 to 30 Hz and 0.9 to 16.9 Hz, depending on the sample. Each measurement was repeated 3 times to ensure consistency and accuracy of the data.

### Strain measurement

To measure strain in the gingival tissue, we first marked the surface with cross signs using a tissue marker. These markings helped in accurately determining the extent of deformation. The lengths of both horizontal and vertical arms of these crosses were meticulously measured before and after the dissection of the gingiva from the bone. For precise length measurement, the samples were positioned at a designated location. Photographs were then taken with a camera fixed at a specific position to ensure consistent imaging. These photographs were subsequently analyzed using ImageJ software to measure the lengths of the marked cross arms. The strain within the tissue was calculated based on the difference in lengths post-excision. All measurements were repeated 3 times. We then computed the average and standard deviation for these measurements.

### OCE system

Our study employed a custom-built swept-source optical coherence tomography (OCT) system, operating at a central wavelength of 1,300 nm and featuring an A-line rate of 43.2 kHz [18]. This system delivered approximately 10 mW of optical power onto the sample. A specialized contact probe, integrating a piezoelectric transducer (PZT) and a 3D printed probe tip, was used to induce elastic waves in the tissue. The probe tip was designed with a radius of curvature of approximately 0.4 mm. For the OCE measurements, we utilized 2 distinct scan patterns. The first pattern involved a frequency sweep ranging from 6 to 16 kHz with 2-kHz intervals, with 172 A-lines per M-scan. For ex vivo porcine tissues, the spatial span for analyzing elastic waves was set at 4 mm at 6 kHz, gradually reducing to 1.65 mm at 16 kHz. In human subjects, scan lengths were adjusted to 6, 5, 4, 3.3, 2.8, and 2.5 mm for frequencies of 6, 8, 10, 12, 14, and 16 kHz, respectively. The second scan pattern was a single tone at 16 kHz, spanning a scan length of 2 mm and involving 360 A-lines per M-scan. Across this setup, a total of 96 transverse points were acquired. The total duration for data collection was approximately 3 s for a frequency sweep and 1 s for a pure tone. The implemented signal processing software effectively mitigates the impact of any potential subject motion during the measurement period. Wave speeds at each frequency (*f*) were determined by performing a Fourier transform of the complex wave amplitude data. After identifying the peak wavenumber (*k*), wave speed was calculated using *ν* = 2*πf*/*k*. The attenuation coefficient was calculated by fitting a linear function to the natural logarithm of the displacement magnitude, from which the 1/*e* propagation distance was derived based on the reciprocal of the attenuation coefficient.

### OCE measurement of porcine samples

Measurements were conducted on various locations of the labial attached gingiva. Each data point presents the average value, and the associated error bar indicates the standard deviation, both derived from 3 independent measurements. Between the measurements, the probe was carefully removed from and then repositioned on to the sample, applying gentle pressure to approximately the same location to ensure consistency.

### OCE measurement of human subjects

The study was conducted in Massachusetts General Hospital (MGH) in accordance with the protocol approved by the Institutional Review Board of MGH and the Mass General Brigham Human Research Office (2023P000338; approval on 07/14/2023). We recruited 4 individuals with healthy periodontal tissues (2 males and 2 females, average age 31.3 ± 2.3 y). Both subjects were free from any physiologically abnormal conditions, including inflammation or gingival recession, and they maintained proper oral hygiene prior to the experiments. Informed consent was obtained prior to the experiments, and all procedures were conducted in accordance with the principles of the Declaration of Helsinki. Disposable dental retractors were used to keep the subjects’ mouths open, ensuring no stress was placed on the temporomandibular joint. During the dehydration tests, subjects’ gingiva was exposed to air for 7 min by keeping the mouth open using the dental retractor. The collected data and corresponding error bars represent the mean ± standard deviation from 5 repeated measurements at each location.

### Statistical analysis

We report the entire data as mean ± standard deviation for the ex vivo porcine gingiva and in vivo human gingiva. The 95% confidence interval, mean, and standard deviation were calculated for the entire data.

## Results

### Characterizations of porcine gingival tissues ex vivo

**Fig. 1.**
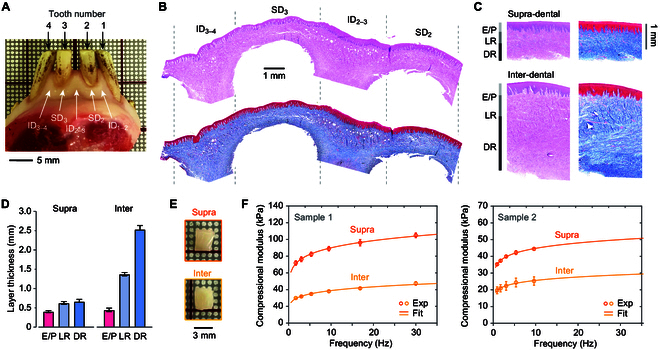
Histological and mechanical analysis of porcine gingiva ex vivo. (A) Photograph showcasing a porcine sample, where various tissue regions are labeled according to the tooth number. (B) Histological cross-sections stained with H&E (top) and Masson trichrome (bottom). (C) Detailed view of selected supra-dental and inter-dental tissue areas. (D) Measured thicknesses of various tissue layers in supra- and inter-dental regions. (E) Images of samples post full-thickness dissection. (F) Graph illustrating the frequency-dependent elastic modulus of gingival tissues from 2 different samples, as measured by DMA.

In our examination of porcine gingival tissue structure and mechanical properties, we utilized fresh porcine mandibles, focusing specifically on the labial gingiva (refer to Fig. 1A). We categorized the regions between teeth as “inter-dental” (ID) and the areas above teeth as “supra-dental” (SD). For simplicity, we numbered the mandibular teeth from right to left as 1 to 4. The subscript next to each label corresponds to tooth numbers; for instance, ID_2–3_ represents the gingival tissue between teeth 2 and 3, while SD_3_ denotes the tissue atop tooth number 3.

Histological examination with H&E stain, along with Masson trichrome stain, disclosed notable differences in collagen network organization between ID and SD areas. These differences also extended to the thickness across different tissue layers (see Fig. 1B). We measured the overall gum thickness to be approximately 3.5 mm in ID regions and about 1.4 mm in SD areas. Although the thickness of the epithelium (E) and papillary layer (P) were similar in both ID and SD regions, marked variations were observed in the loose reticular layer (LR) and the dense reticular layer (DR), as illustrated in Fig. 1C and D. In the LR, collagen fibers appeared thinner and more densely packed compared to those in the DR. Furthermore, collagen fiber orientation was more randomized in the LR, in contrast to the more parallel alignment relative to the surface in the DR.

For establishing baseline data on tissue stiffness, we employed a dynamic mechanical analyzer (DMA). We measured the compressional moduli of bulk tissue sections extracted from both ID and SD regions (depicted in Fig. 1E). Representative samples were analyzed with a displacement amplitude of 30 μm across a frequency range from 1.7 to 30 Hz, with results presented in Fig. 1F. The minimal displacement applied ensures that the tissue undergoes linear deformation. Observations revealed that the compressional modulus is frequency-dependent, displaying a sublinear increase with frequency, a behavior typical of viscoelastic soft tissues. Notably, there were significant variations in the elastic modulus between samples from different animals, with disparities up to a factor of up to 2. However, for samples extracted from the same animals, the average compressional elastic modulus of SD tissues was consistently about 2 times higher than that of the ID tissues. Integrating our structural and mechanical data, we infer that the discernible mechanical distinctions in bulk tissues are largely attributable to the collagen network and anatomical morphology of gingiva.

### OCE measurement of porcine gingiva ex vivo

**Fig. 2.**
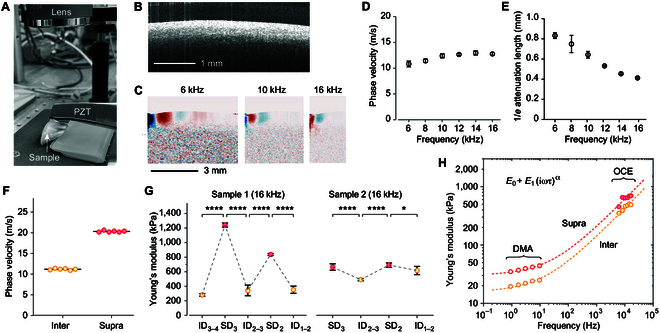
OCE measurements of porcine gingiva tissues ex vivo. (A) Setup photograph showing a PZT probe utilized in OCE. (B) Representative OCT image of porcine gingival tissue. (C) Displacement profiles of elastic waves at various frequencies captured by OCE. (D) Typical curve showing wave velocity dispersion. (E) Graph depicting 1/*e* attenuation lengths as a function of frequency. (F) Demonstration of repeatability of wave velocity measurements at 2 distinct locations. (G) Young’s modulus values of various positions in 2 different samples. (H) Comparison of frequency-dependent elastic modulus as measured by DMA and OCE. Dashed lines indicate theoretical fitting based on the fractional viscoelastic model, with parameters for supra-dental tissues (*E*_0_ = 30 kPa, *α* = 0.5, and *E*_1_(2*πτ*)*^α^*= 6 kPa) and for inter-dental tissues (*E*_0_ = 15.6 kPa, *α* = 0.5, and *E*_1_(2*πτ*)*^α^* = 4.2 kPa).

We employed an OCE system to measure the mechanical properties of porcine gingiva. This system used a PZT probe to generate elastic waves in the tissue with submicron displacement amplitudes, typically within a frequency range of 2 to 20 kHz (Fig. 2A). The imaging depth was generally limited to 1 mm. Due to limited contrast in light scattering between different tissue layers, structural OCT images could not distinctly delineate tissue structures, as shown in Fig. 2B. By analyzing the vibration of the tissue at the driving frequency of the PZT, OCE signal processing yields images of the elastic wave. Representative OCE images obtained at 6, 10, and 16 kHz with the PZT probe are presented in Fig. 2C. We determined the wavelength and 1/*e* propagation length of these waves by analyzing displacement waveforms in the Fourier transform domain [16]. Figure 2D and E displays representative data across a frequency range from 6 to 16 kHz, where we observed a modest increase in wave velocity with frequency. The wavelengths measured were 1.8, 1.44, 1.24, 1.06, 0.93, and 0.8 mm at frequencies of 6, 8, 10, 12, 14, and 16 kHz, respectively. The 1/*e* attenuation lengths showed an exponential decrease with frequency. These wavelength and propagation length measurements provide an estimate of OCE’s spatial resolution. At 16 kHz, the spatial resolution was found to be less than 1 mm. The chosen frequency range of 6 to 16 kHz is optimal for gingival characterization for several reasons. Frequencies below 6 kHz result in elastic wavelengths too long to differentiate between SD and ID regions, and the low-frequency waves may extend to underlying bone and tooth structures, leading to inaccuracies in gingival measurement. Conversely, frequencies above 16 kHz decrease both displacement amplitude and propagation distance nonlinearly, reducing measurement accuracy.

Figure 2F demonstrates the repeatability of velocity measurements at 16 kHz on the same tissue location, also highlighting significant regional differences. The observed factor of 2 difference is consistent with the previous results obtained using DMA. When the PZT probe’s contact point was shifted by 0.5 mm, variations of no more than 10% were observed. The shear modulus (*G*) was calculated from the wave velocity (*v*) using a formula,

$$G=A\rho v^{2}$$ (1)

where *ρ* (~1,100 kg/m^3^) is the mass density of tissue, and the coefficient *A* is equal to 1 for pure shear waves and 1.096 for Rayleigh surface waves in isotropic, nonstrained materials. The OCE elastic waves at 16 kHz can be considered surface waves as their wavelengths (~0.8 mm) are shorter than the thickness of tissue. For incompressible, isotropic materials, Young’s modulus (*E*) relates to shear modulus via *E* = 3*G*. Using these formulas, we derived Young’s modulus values of gingival tissues, as shown in Fig. 1G. The data reveal mechanical differences between ID and SD regions, along with variations between samples. Figure 2H compares the compressional modulus measured using DMA with the equivalent Young’s modulus obtained using OCE. The data fitting reasonably aligns with the formula *E* = *E*_0_ + *E*_1_(*iωτ*)*^α^*, based on the fractional Kelvin–Voigt model, which is one of the standard models used to interpret the behaviors of viscoelastic materials [19]. Here, *E*_0_ represents the purely elastic component, while *E*_1_(*iωτ*)*^α^* describes the viscoelastic component (the exponent *α* ranges from 0 for a spring to 1 for a dashpot). The best curve fit was achieved using *E*_0_ = 30 kPa, *α* = 0.5, and *E*_1_(2*πτ*)*^α^* = 6 kPa for supra-dental tissues and *E*_0_ = 15.6 kPa, *α* = 0.5, and *E*_1_(2*πτ*)*^α^* = 4.2 kPa for inter-dental tissues. An exponent *α* ranging from 0.5 to 0.75 has been observed in cells and polymers which was attributed to the behavior of crosslinked polymer networks [20,21].

The correlation of measurement data with the viscoelastic model underscores the mechanistic foundation of OCE measurements. Traditional mechanical tools, including DMA and palpation-based techniques, typically assess tissue properties through quasi-static or low- frequency deformations. By contrast, OCE characterizes the elastic modulus in the kilohertz range. This kilohertz modulus is inherently linked to the low-frequency modulus via the tissue’s viscoelastic properties. It is important to emphasize, however, that OCE’s goal is not to estimate the low-frequency elastic modulus. Instead, its primary aim is to elucidate the structural and physiological states of gingival tissues by examining their mechanical properties.

### Analysis of in situ strain and tissue isotropy

**Fig. 3.**
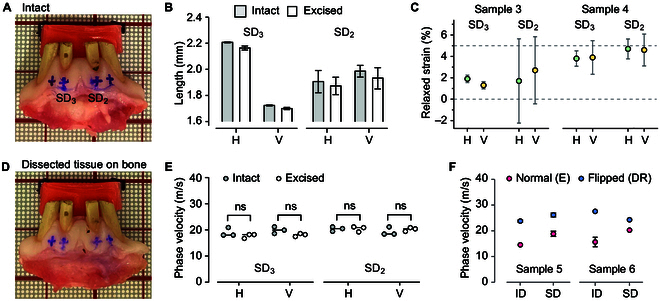
Assessing isotropy and linearity of porcine gingiva tissue in situ. (A) Illustration of cross signs marked on the tissue prior to dissection. (B) Comparative measurements of the horizontal (H) and vertical (V) arms of the cross marks before and after excision at 2 different locations. (C) Calculated magnitudes of relaxed strain post-excision in both horizontal and vertical directions. (D) Image depicting the dissected tissue repositioned on the bone for post-excision OCE measurements. (E) Measured wave velocities in horizontal and vertical directions, both before and after excision. (F) Wave velocity measurements on dissected samples in normal configuration (with the PZT probe on the epithelial surface) and flipped configuration (with the probe on the dense reticular surface), conducted on 2 separate samples.

Gingival tissues, like other soft tissues, can exhibit anisotropic properties. Particularly, the attached gingiva on the bone is likely under tension and tensile strain. This strain influences the tissue’s elastic modulus due to the nonlinear characteristic of collagen fibers, subsequently affecting wave velocity. Additionally, tension can impart an extra restoring force, further impacting wave velocity. Utilizing the acoustoelastic effect [22], we find that Eq. 1 is valid with *A* ≈ 1.1 − 2.5*ε* where *ε* denotes small strain (derived for surface waves in a neo-Hookean material). To measure strain *ε* in situ for porcine gingiva, we marked the tissue with cross-patterns of ink (Fig. 3A) and excised the full thickness of the attached gingiva using a scalpel. By comparing the lengths of the cross marks before and after excision (Fig. 3B), we determined the in situ strain to be approximately 3.0 ± 1.5% for the horizontal direction and 3.1 ± 1.6% for the vertical direction (Fig. 3C). Therefore, *A* = 1 is a good approximation to describe gingival tissues in situ. We used this value throughout this study.

After positioning the excised tissues back onto the bone (Fig. 3D), we measured wave velocities at 16 kHz using OCE. The results showed no significant differences from the velocities originally measured on the intact gingiva prior to surgical dissection (Fig. 3E). Additionally, there was no notable difference in wave velocities between the horizontal and vertical directions. These findings suggest that gingival tissues in situ can be considered nearly transversely isotropic and behave as linear materials. In addition, we conducted tensile strain–stress tests on porcine gingival tissues, which revealed near-linear behavior for small strains up to 5%. Beyond this range, at larger stains, we observed a nonlinear increase in the elastic modulus, a characteristic typically exhibited by soft tissues containing collagen fibers.

To explore tissue heterogeneity along the depth, we conducted velocity measurements with the PZT probe placed on the side facing the DR, as opposed to the normal epithelial surface. The wave velocities at 16 kHz, measured from the DR surface, were consistently higher than those from the epithelial surface (Fig. 3F). This observation aligns with the expectation that Rayleigh elastic waves predominantly localize their energy within the surface up to a wavelength deep. Therefore, the propagation velocity is predominantly determined by the elastic modulus of the surface region [23]. With a wavelength of approximately 0.8 to 1.4 mm at 16 kHz, the wave energy is concentrated in the epithelium and papillary layer (E/P) when probed normally, while it is localized within the DR region in the flipped samples. This finding confirms the higher elastic modulus of the DR region compared to the E/P region.

### OCE measurement of human gingiva in vivo

**Fig. 4.**
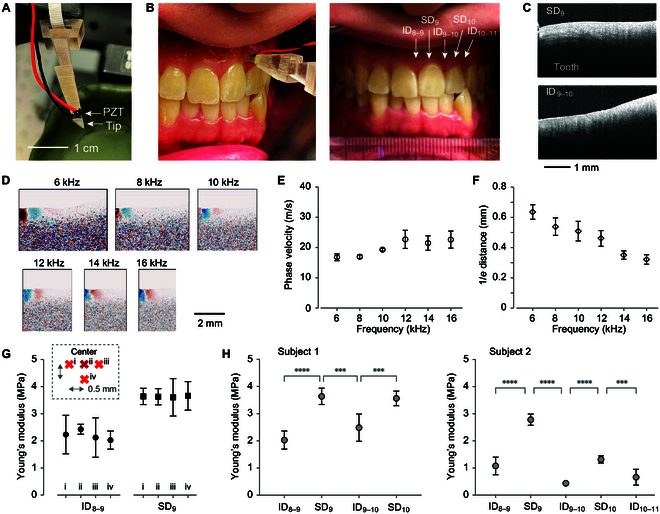
In vivo OCE measurements of human gingiva. (A) A photograph of the modified probe used for in vivo OCE. (B) Photographs showing the gingiva of a human subject and the contact PZT probe in place, including labels for different tissue regions. (C) OCT images representing human gingiva in both supra-dental and inter-dental areas. (D) Profiles of elastic wave displacement at various frequencies as captured by OCE. (E) A typical curve depicting wave velocity dispersion. (F) A typical curve showing the 1/*e* attenuation distance. (G) Analysis of the repeatability and regional variability of Young’s modulus in distinct tissue areas. The inset illustrates 4 probe contact points used to assess regional variations within small 0.5-mm by 0.5-mm areas in both inter- and supra-dental regions. (H) Elastic moduli measured at various positions from 2 different subjects.

To extend the application of OCE to human subjects, we recruited 4 healthy individuals in their early 30s and developed a PZT probe with an ergonomic design for comfortable usage (Fig. 4A). This probe was applied to various positions on the labial attached gingiva, as depicted in Fig. 4B, and elastic waves were launched along the horizonal direction. We used the standard tooth numbering system, ranging from 1 to 16 for upper teeth and 17 to 32 for lower teeth. To minimize dehydration, OCE was performed immediately following the application of a dental retractor.

Structural OCT imaging revealed the full thickness of the gingiva in relatively thinner SD regions but was less effective in the thicker ID regions (Fig. 4C). The OCE images displaying elastic propagation (Fig. 4D) exhibited propagation speeds and attenuation similar to those observed in porcine tissues. As shown in Fig. 4E and F, the wave velocity modestly increased with frequency, and the propagation distance decreased inversely with frequency. This behavior aligns with the viscoelastic properties of tissue. With a speed of 22.3 m/s at 16 kHz, we measured the wavelength to be 1.4 mm, resulting in a shear modulus of 547 kPa and a calculated Young’s modulus (*E*) of 1.64 MPa.

Figure 4G presents Young’s moduli measured at 4 different locations, spaced 0.5 mm apart, within the ID_8–9_ and SD_9_ regions of the same subject. The error bar for each data point represents the standard deviation from 5 repeated measurements, which involved retracting and recontacting the probe at the same target location. The repeatability of in vivo measurements was lower compared to ex vivo measurements, attributable to minor shifts in probe positioning. Despite this, the spatial variation within a 0.5-mm by 0.5-mm region remained within the measurement uncertainty. However, significant differences were observed between the ID and SD regions across various human subjects, as shown in Fig. 4H. Subjects reported no pain during the measurements.

### Mechanical changes in various gingival tissue conditions

**Fig. 5.**
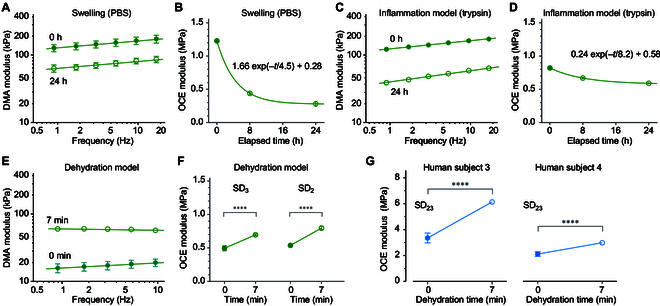
Elastic modulus measurements under varied physiological conditions. (A) Comparison of the elastic modulus of porcine tissue measured by DMA, both in its native state (0 h) and after swelling in PBS for 24 h. (B) Time-lapse OCE monitoring of changes in the elastic modulus induced by swelling. (C) DMA measurements of porcine gingiva intact (0 h) and after 24 h of trypsin treatment. (D) Time-lapse OCE monitoring of changes in porcine tissue elastic modulus during trypsin treatment. (E) DMA measurements comparing the elastic modulus of porcine tissue in its natural state (0 min) and after 7 min of natural drying by air exposure. (F) OCE measurements of porcine tissue pre- and post-drying. (G) OCE measurements documenting changes in the elastic modulus of human gingiva in 2 subjects during a 7-min dehydration period caused by air exposure. The OCE moduli were measured at a frequency of 16 kHz.

In exploring the utility of OCE for characterizing different physiological states of gingival tissue, we conducted several experiments. First, to mimic inflammation-induced swelling, we placed PBS-immersed gauze on ex vivo porcine samples. Using DMA on various samples, both with and without PBS treatment, we observed a significant reduction in elastic modulus values after 24 h (Fig. 5A). OCE allowed us to track the change in elastic modulus over time on the same samples, displaying an exponential decay curve to a swelling equilibrium with a time constant of approximately 4.5 h (Fig. 5B).

Next, we simulated inflammation-induced degradation of collagen fibers using trypsin treatment. Trypsin, a serine proteinase known to hydrolyze collagen fibers [24–27], is expected to decrease the elastic modulus [28,29]. Porcine samples treated with trypsin-soaked gauze for 24 h exhibited notably lower elastic moduli, as measured by DMA (Fig. 5C). Time-lapse OCE monitoring revealed a gradual decrease in elastic modulus over time, with an estimated time constant of around 8.2 h.

Third, we examined a dehydration model by exposing tissues to air for 7 min. For ex vivo porcine samples, DMA measurements indicated a significant increase in elastic modulus post-dehydration (Fig. 5E). OCE corroborated this dehydration-induced stiffening (Fig. 5F). Finally, we applied this dehydration model to human gingiva in vivo. OCE measurements were taken on human subjects immediately and 7 min after wearing a dental retractor. The results consistently indicated an increase of elastic modulus after 7 min of dehydration (Fig. 5G).

Overall, our findings underscore the responsiveness of mechanical properties to various tissue conditions and highlight the potential of OCE as a sensitive tool for detecting these induced changes in clinical settings.

## Discussion

In this study, we have provided a quantitative characterization of gingival tissue under various conditions using high-frequency OCE. Our findings reveal distinct stiffness variations in the gingiva, correlating with its anatomical morphology around the teeth. Notably, supra-dental gingiva exhibited greater stiffness compared to inter-dental gingiva, a result corroborated by both DMA and OCE. Additionally, we demonstrated the viscoelastic, nearly linear, and transverse-isotropic properties of gingival tissue in situ, validating the reliability of OCE measurements. OCE was further optimized for use in human subjects, yielding consistent results. We also explored the impact of tissue hydration and collagen degradation on gingival stiffness, suggesting potential applications of OCE in detecting physiological changes in a clinical setting. We observed that both hydration and collagen degradation led to a decrease in gingival stiffness, while dehydration caused an increase.

Our research demonstrates the feasibility and safety of OCE for in vivo biomechanical characterization of gingival tissues. Prior attempts at noninvasive gingival measurement were limited to optical imaging techniques, which fell short of providing mechanical insights [30–33]. A stride was made in a recent study employing OCE on rabbit gingiva to gauge stiffness, marking a preliminary foray with limited scope [34]. Herein, we extend the horizon by presenting the inaugural noninvasive, in situ characterization of human gingival tissue using OCE, marking a first in applying this technique to human gingiva.

The high-frequency OCE presented in this study offers superior resolution over conventional mechanical testing, ultrasound, and magnetic resonance imaging. While traditional mechanical testing is limited to assessing the bulk properties without spatial detail, OCE achieves an elastography resolution of approximately 1 mm at 16 kHz in gingival tissues, allowing for the detection of local biomechanical variations. High-frequency waves employed in OCE afford higher resolution compared to ultrasound and magnetic resonance imaging, and the high optical phase sensitivity of OCE to microscopic vibrations facilitates a more precise shear modulus measurement.

Although porcine and human gingiva share anatomical similarities [35], subtle variances could influence the interpretation of results. For instance, human gingiva displayed higher stiffness and thus lower spatial resolution in OCE. Additionally, variations in thickness atop the teeth between porcine and human samples could influence shear modulus measurements, potentially affected by the bone beneath. Nonetheless, the consistent phase velocities observed (Figs. 2D and 4E) imply that these thickness differences were not substantial enough to impact sensitivity difference across porcine and human tissues significantly.

OCE’s potential is vast, with room for further refinement to enhance accuracy and clinical applicability. Expanding the frequency range beyond 16 kHz could improve resolution and enable the determination of layer-specific mechanical properties [23,36]. Designing smaller probes could allow access to deeper oral regions and a broader spectrum of tissues. Advances in measurement speed could reduce errors from patient movement and time-sensitive variables like hydration changes.

The oral mucosa, including the gingiva, covers the majority of the oral cavity’s surface, encompassing the teeth and underlying bone, thereby providing adequate biological barriers and mechanical protections. Its major molecular component, collagen fibers [4], has been associated with the normal healthy and diseased states of the gums. Gingival inflammation, one of the most common diseases, can cause the degradation of collagen fibers [4,8]. The oral squamous cell carcinoma, which accounts for 96% of all oral cancers, induces the loss and disruption of the collagen fiber matrix [37,38]. The collagen fiber orientation and density are important indicators of healing after dental implant surgeries [39,40] and gingival surgeries [41,42]. During and following orthodontic treatments, the collagen fiber orientation affects tooth movement and rotation [43,44]. These collagen fibers are primarily responsible for the specific mechanical properties of the tissue. It has been established that the organization [5] and cross-linking density of collagen fibrils [6] are closely related to the elastic moduli of tissue [7]. Tissue hydration and swelling are also well known to influence tissue elasticity [9]. Therefore, the microstructural changes of collagen fibers in different medical conditions are expected to be reflected in the bulk mechanical properties of the gingiva. Considering the close relationship between tissue status and mechanical properties, a deeper understanding of tissue elasticity may provide novel insights into biomechanics-based monitoring of gingival health and diseases. In this endeavor, the ability to measure gingival tissue elasticity in vivo would be highly useful.

Although preliminary, our study paves the way for utilizing OCE in periodontal diagnosis and beyond. Envisioning an OCE system alongside dental chairs, practitioners could assess tissue stiffness with millimeter-scale resolution in real time using a handheld probe on a flexible arm. Longitudinal studies could monitor gingival stiffness changes due to pathological conditions, aiding in diagnosis and treatment strategies. OCE could also support oral surgery, enhancing biopsy or prediagnosis of various oral diseases. This technology might also find applications in oral tissue engineering, particularly in the synthesis of collagen-fiber-based biomaterials [45–47]. OCE can provide patient-specific stiffness data, potentially optimizing surgical grafting materials for reconstructive surgeries [48–51].

## Ethical Approval

The study was conducted in MGH in accordance with the protocol approved by the Institutional Review Board of MGH and the Mass General Brigham Human Research Office (2023P000338; approval on 2023 July 14).

## Data Availability

The datasets used and/or analyzed during the current study are available from the corresponding author on reasonable request.
